# Editorial: Role of Iron in Bacterial Pathogenesis

**DOI:** 10.3389/fcimb.2018.00344

**Published:** 2018-10-16

**Authors:** Susu M. Zughaier, Pierre Cornelis

**Affiliations:** ^1^Department of Basic Medical Sciences, College of Medicine, Qatar University, Doha, Qatar; ^2^Microbiology Unit, Department of Bioengineering Sciences, Vrije Universiteit Brussel and VIB Department of Structural Biology, Brussels, Belgium

**Keywords:** iron depletion, virulence factors, host defense against pathogenic bacteria, iron-regulated genes, siderophore

Iron is the fourth-most abundant element on the earth, and it is needed by most organisms, including bacteria. It exists in two oxidation states, Fe^2+^ and Fe^3+^, and is involved in many oxido-reduction reactions (Andrews et al., 2013). Ferric iron (Fe^3+^) is the dominant form in oxygenated environments and has a very low solubility, which presents a problem for microorganisms with an aerobic lifestyle (Andrews et al., 2013). Conversely, in anaerobic environments or in microaerobic conditions at low pH, the soluble ferrous iron (Fe^2+^) is the most abundant form (Andrews et al., 2003). Bacterial pathogens face a problem because free iron is not available since it is bound to heme or by circulating proteins such as transferrin or lactoferrin (Finkelstein et al., 1983; Cornelissen and Sparling, 1994). Pathogens use different strategies to obtain iron from the host, via the production of extracellular Fe^3+^-chelating molecules termed siderophores (either their own or produced by other microorganisms), the uptake of heme, and the uptake of Fe^2+^ (Feo system) (Andrews et al., 2013). A single pathogen can adapt its iron-uptake strategy in response to the type of infection (acute or chronic) and the availability or lack of ferrous iron (Cornelis and Dingemans, 2013). In this issue, several authors present several facets around iron uptake in different bacterial pathogens. *Yersinia pestis* produces the yersiniabactin siderophore under aerobic conditions and the Feo Fe^2+^ uptake system under microaerobic conditions (Fetherston et al., 2012). The *feo* operon of *Y. pestis* is peculiar since it is repressed by Fe via the Fur repressor only under microaerobic, but not under aerobic conditions, unless the promotor region is truncated. The other facet of the host-pathogen battle for iron is the host response to the bacterial pathogen. As shown, again for *Y. pestis*, a live vaccine induces an iron nutritional immunity via the production of hemopexin and transferrin iron-binding proteins.

Some pathogenic bacteria infect fish, such as *Vibrio anguillarum* and *Photobacterium damselae*, both belonging to the Vibrionaceae. Citrate is probably the simplest siderophore and is produced by the citrate synthase and excreted by *P. damselae* strains unable to produce the vibrioferrin siderophore, thus establishing a link between the cellular metabolism and iron uptake. In their review article, Li and Ma describe the ways by which different *V. anguillarum* pathogenic strains take up iron either via the production of siderophores (anguibactin, vanchrobactin), uptake of xenosiderophores enterobactin or ferrichrome, or uptake of heme or ferrous iron. *Burkholderia* represents a genus of β-proteobacteria with 90 species, including the *B. cepacia* complex (BCC), which cause infections in the lungs of patients with cystic fibrosis and *B. pseudomallei*, which causes meloidiosis. Butt and Thomas reviewed the different iron-uptake strategies of these highly adaptable bacteria, including the production of siderophores (ornibactins, cepaciachelin, pyochelin, malleobactin), the uptake of heme and of ferrous iron. *Francisella tularensis* is the causative agent of tularemia and able to replicate in macrophages. *F. tularensis* can take up the siderophore rhizoferrin, but relies on the Feo system for the uptake of ferrous iron. The uptake of rhizoferrin does, however, not need the TonB protein as in other bacteria, while the uptake of Fe^2+^ involves an outer membrane protein termed FupA, which is also unusual. Inhibition of the uptake of iron by bacteria involves, among other approaches, the use of gallium–protoporphyrin IX as shown in the case of *Pseudomonas aeruginosa*. The GaPPX is a heme analog that can be taken up via outer membrane heme receptors, inhibiting the growth of *P. aeruginosa* under conditions of iron limitation. Once in the cell, GaPPX was shown to target the aerobic respiration.

Bacterial pathogens sense iron-limiting conditions and respond accordingly by upregulating iron-acquisition mechanisms and virulence genes (Zughaier et al., 2014). Mouméne et al. report that the intracellular bacteria *Ehrlichia ruminantium* upregulates the type 4 secretion system (T4SS) and virulence genes under iron depletion via the newly identified master regulatory protein ExrR. Ferric uptake regulator (Fur) is a transcription factor that upregulates virulence factors in bacteria during iron depletion. Guo et al. used an unmarked gene deletion system to investigate the role of Fur in the virulence of *Riemerella anatipestifer*, an avian pathogen. Using RNA-seq analysis, they determined fur-regulated genes and identified putative fur-binding sequences. Their study further demonstrated that deleting the *fur* gene led to a reduction of virulence *in vivo*. In response to infection, the host limits the bioavailability of iron by upregulating expression of hepcidin, the master iron-regulating hormone, which limits iron uptake from the gut and retains iron in macrophages. Nairz et al. investigated the role of dietary iron enrichment in host-pathogen interactions during *Salmonella typhimurium* infection in mice with hereditary hemochromatosis (genetic *Hfe*-deficiency) compared to wild type. They observed that *Salmonella* infection induced hepcidin and hypoferremia in an Hfe-independent manner. However, iron overload increased the bacterial load in mice. Further, *Salmonella* infection in mice responded to iron-depleting conditions in the host by upregulated iron-acquisition genes.

Malhotra et al. report that *Mycobacterium tuberculosis (M. tb)* utilizes its highly conserved glycolytic enzyme GAPDH to acquire iron from the host by binding to lactoferrin with high affinity. *M. tb* sequesters iron from lactoferrin bound to GAPDH on the surface of bacteria. Sharma and Bisht provide a perspective on the role of iron-storing proteins in the emergence of antibiotic resistance. Based on their previous observation that iron-storing proteins, bacterioferritin (Rv1876) and ferritin (Rv3841), were overexpressed in aminoglycosides-resistant isolates of *M. tb*, they used a computational approach (STRING analysis) to predict protein partners that interact with bacterioferritin and ferritin. Among the identified partners is the hypothetical transmembrane protein Rv1877, which is predicted to be involved in drug resistance; therefore, Rv1877 may be a potential drug discovery target.

## Author contributions

All authors listed have made a substantial, direct and intellectual contribution to the work, and approved it for publication.

### Conflict of interest statement

The authors declare that the research was conducted in the absence of any commercial or financial relationships that could be construed as a potential conflict of interest.
